# Draft Genome Sequence of a Multidrug-Resistant Escherichia coli Sequence Type 1193 Pandemic Clone Isolated from Wastewater in Austria

**DOI:** 10.1128/MRA.00762-21

**Published:** 2021-09-16

**Authors:** Adriana Cabal, Nadine Peischl, Gerhard Rab, Anna Stöger, Burkhard Springer, Jasmin Sucher, Franz Allerberger, Werner Ruppitsch

**Affiliations:** a Austrian Agency for Health and Food Safety, Vienna, Austria; b Institute of Hydraulic Engineering and Water Resources Management, Vienna University of Technology, Vienna, Austria; c Institute for Land and Water Management Research, Federal Agency for Water Management, Petzenkirchen, Austria; d Austrian Agency for Health and Food Safety, Graz, Austria; e Department of Biotechnology, University of Natural Resources and Life Sciences, Vienna, Austria; Montana State University

## Abstract

Extraintestinal Escherichia coli sequence type 1193 (ST1193) is an important source of fluoroquinolone resistance, which has emerged in recent years. We report the first draft genome sequence and annotation of a multidrug-resistant E. coli ST1193 strain obtained from a wastewater treatment plant in Austria.

## ANNOUNCEMENT

Escherichia coli sequence type 1193 (ST1193) was recently described as an extraintestinal (exPEC) fluoroquinolone-resistant (FQ^R^) pandemic clone (1–5). ST1193 strains can cause extraintestinal disease in humans (1) and companion animals (6, 7), and they have been isolated from environmental sources (8). Features of this clone are the inability to ferment lactose and the production of extended-spectrum β-lactamases (ESBLs), in particular CTX-M.

In 2020, an inlet water sample was collected from a biological wastewater treatment plant in Austria. The sample was filtered through a 0.45-μm pore filter (Sartorius Biosart, UK). The filter was subsequently placed in 9 ml thioglycolate medium (Oxoid, Germany) and incubated for 24 h at 37°C. An aliquot (100 μl) was plated onto ReadyPlate CHROM CCA ISO9308 agar (Merck, Germany). Random violet/blue colonies were subcultivated on blood agar (bioMérieux, Marcy-l’Étoile, France) for 24 h at 37°C and identified as E. coli using matrix-assisted laser desorption ionization–time of flight mass spectrometry (MALDI-TOF MS) (Brucker, Germany). Whole-genome sequencing (WGS) was performed as previously described (9). Briefly, high-molecular-weight (HMW) DNA was extracted from the isolates using the MagAttract HMW DNA kit (Qiagen, Hilden, Germany). Genomic libraries were prepared using the Nextera XT library preparation kit (Illumina, San Diego, CA), followed by 2 × 250-bp sequencing on the Illumina MiSeq platform. Default parameters were used for all software unless otherwise specified. The quality of the raw reads was checked using FastQC v0.11.9 and adapter sequences were removed using Trimmomatic v0.36. The last 10 bp of each sequence and those sequences below a quality score of 20 were trimmed. The reads were assembled using SPAdes v3.11.1. Ridom SeqSphere v7.7.5 was used for WGS analysis, including multilocus sequence typing (MLST) and core genome MLST (cgMLST) using E. coli EnteroBase schemes. Plasmid presence, serotype, fimbria types, antimicrobial resistance genes, and virulence genes were identified using Center for Genomic Epidemiology (CGE) server tools.

The genome assembly of one E. coli isolate contained 5,182,660 bases with 150 contigs, a contig *N*_50_ value of 150,776 bp, a GC content of 50.61%, and a genome coverage of 75×. The NCBI Prokaryotic Genome Annotation Pipeline (10) yielded a total of 5,295 genes, including 4,982 coding genes, 3 rRNAs, 92 tRNAs, 6 noncoding RNAs, and 189 pseudogenes.

The isolate was ST1193, serotype O75:K1:H5, and *fim*H64. It carried mutations in *gyrA* (S83L, D87N), *parC* (S80I, E62K), and *parE* (L416F), conferring fluoroquinolone resistance, and harbored *bla*_CTX-M-55_ and *bla*_TEM-1_, among other resistance genes (Table 1). The isolate was resistant when tested on Mueller-Hinton (bioMérieux) agar using Etest strips (bioMérieux) to ampicillin (MIC, 256 μg/ml), cefotaxime (MIC, 32 μg/ml), ciprofloxacin (MIC, 32 μg/ml), and gentamicin (MIC, 32 μg/ml) and borderline for cefepime (MIC, 4 μg/ml). A Col156 plasmid, widely distributed among ST1193 (1), was detected using PlasmidFinder. The *bla*_CTX-M-55_ gene was not carried in any of the plasmids reported by PlasmidFinder, but the contig carrying this gene presented 99.79% identity to a Klebsiella pneumoniae plasmid (GenBank accession no. CP064254.1) when using BLAST (11). Several virulence genes (VGs) were detected (Table 1).

**TABLE 1:** Characteristics of the Austrian ST1193 *E. coli* clone Ec-510423-20^*a*^

ARGs	VGs	Plasmids
*bla*_CTX-M-55_, *bla*_TEM-1B_, *aac(3)-IId*, *aph(3*″*)-Ib*, *aph(6)-Id*, *aadA5*, *dfrA17*, *erm*(B), *gyrA* (S83L, D87N), *parC* (S80I, E62K), *parE* (L416F), *mdf*(A), *mph*(A), *qacE*, *sitABCD*, *sul1*, *sul2*	*chuA*, *fyuA*, *iha*, *irp2*, *iucC*, *iutA*, *kpsE*, *kpsMII_K1, neuC*, *ompT, papA_F43*, *sat*, *senB*, *sitA*, *terC*, *traT*, *usp*, *vat*, *yfcV*	Col(BS512), Col156, IncB/O/K/Z, IncFIA/IncFIB

a ARGs, antimicrobial resistance genes; VGs, virulence genes.

cgMLST-based comparisons of our isolate with publicly available genomes revealed that a neonatal meningitis O75:H5 E. coli strain (strain MCJCHV-1; GenBank accession no. CP030111) (12) negative for CTX-M and TEM was the closest (25 allelic differences) to our ST1193 isolate, followed by a CTX-M-27-harboring Irish isolate from seawater, also collected in 2020 (PubMLST ID 402).

### Data availability.

This whole-genome shotgun project has been deposited in DDBJ/ENA/GenBank under accession no. JAHTLG000000000.1 and BioProject accession no. PRJNA743849. The version described in this paper is the first version. The raw sequence reads have been deposited in the Sequence Read Archive (SRA) under accession no. SRR15042476.
